# Depressive symptoms and chronotypes of elderly nursing home residentes: A case management study

**DOI:** 10.1590/1980-57642020dn14-020010

**Published:** 2020

**Authors:** Evany Bettine de Almeida, Thais Bento Lima-Silva, Luiz Menna-Barreto

**Affiliations:** 1 Programa de Pós-graduação em Estudos Culturais da Escola de Artes, Ciências e Humanidades da Universidade de São Paulo (EACH-USP), São Paulo, SP, Brazil.; 2 Curso de Graduação em Gerontologia da Escola de Artes, Ciências e Humanidades (EACH-USP), São Paulo, SP, Brazil.; 3 Grupo de Neurologia Cognitiva e do Comportamento (GNCC) da Faculdade de Medicina da Universidade de São Paulo, São Paulo, SP, Brazil.

**Keywords:** sleep, depression, institutionalization, temporal conflict, temporal clash, sono, depressão, institucionalização, conflito temporal, choque temporal

## Abstract

Institutionalization potentiates the tendency for specific sleep disturbances which occur with aging, besides negative health consequences such as increased rate of depressive symptoms.

**Objective:**

To compare sleep profile and depressive symptoms in elderly nursing home residents, highlighting gender differences.

**Methods:**

A quantitative descriptive study of 29 elderly from two different nursing homes was conducted. A sociodemographics questionnaire, Sleep Diary, Morningness-Eveningness Questionnaire and the 15-item Geriatric Depression Scale were applied. Data were analyzed using descriptive statistics, Student’s *t-*test and the Mann-Whitney U-test.

**Results:**

The sample comprised individuals that were predominantly female (72%), aged 80-90 years (48%), widowed (66%) and low-educated (83%). The women were found to sleep and awake later than the men. Regarding chronotypes, the women were classified as evening types and men as intermediate/indifferent types. Most of the elderly exhibited symptoms of major depression (48%). Compared to men, women had more depressive symptoms in both dysthymia and major depression categories.

**Conclusion:**

No significant differences were evident on comparisons of sleep profile and depressive symptoms, but elderly with the intermediate chronotype scored lower on the depressive symptoms scale.

## RESUMO.

A institucionalização potencializa a tendência às alterações específicas que ocorrem no sono com o envelhecimento, além de consequências negativas para a saúde como o aumento da prevalência de sintomas depressivos.

**Objetivo:**

Comparar o perfil do sono com os sintomas depressivos de idosos residentes em instituições de longa permanência para idosos.

**Métodos:**

Trata-se um estudo descritivo e quantitativo, desenvolvido 29 com idosos residentes em duas diferentes Instituições de Longa Permanência para Idosos. Foram utilizados um questionário de informações sociodemográficas, Diário do Sono, Questionário para identificação de Indivíduos Matutinos e Vespertinos e Escala de Depressão Geriátrica versão de 15 itens. Os dados foram analisados por meio de uma estatística descrita, Teste t de *Student* e teste U de *Mann-Whitney.*

**Resultados:**

Houve predomínio de mulheres (72%%), idade entre 80 a 90 anos (48%), viúvas (66%) e com baixa escolaridade (83%). Observou-se que as mulheres dormiram e acordaram mais tarde comparados aos homens. Quanto ao cronotipo, as mulheres foram classificadas como vespertino e os homens como intermediários/ indiferentes. A maioria dos idosos apresentou sintomas de depressão maior (48%), quando comparado aos homens, as mulheres manifestaram mais sintomas depressivos, tanto na categoria distimia quanto depressão maior.

**Conclusão:**

Conclui-se que não houve diferença significante nas comparações entre o perfil do sono e sintomas depressivos, porém os idosos com o cronotipo intermediário apresentaram menor escore na escala de sintomas depressivos.

Population aging is a global phenomenon resulting from the increase in life expectancy of the elderly population^1^ and is accompanied by a predominance of chronic disease, increasing health service demands and the need for specific public policies. Concomitantly, contemporary changes in living arrangements have contributed to a deficit in support and family care for the elderly, leading to a rise in demand for nursing homes and preferably without the stigmatized view that these places awakened in society.^2^^,^ 3

Nursing homes are public or private institutions providing residential accommodation for individuals aged ≥60 years who are independent with no income and/or family, or dependent and in need of long-term care.^4^ However, the changes inherent to aging and preexisting diseases may be aggravated by institutionalization, having deleterious effects on the health of elderly residents, especially in the form of sleep disorders.^5^

Institutionalization can increase the tendency for specific sleep disturbances that occur with aging, particularly sleep fragmentation with frequent nocturnal awakenings, which may cause daytime sleepiness.^6^^-^ 8 The literature indicates that poor sleep quality can be associated with negative health consequences, such as an increased risk of falls,^9^ cognitive impairment^10^^,^ 11 and depressive symptoms.^12^^,^ 13

A study of 116 institutionalized elderly in Turkey found that half of the participants had poor sleep quality. Variables associated with poor sleep quality included gender (female), marital status, period institutionalized, presence of chronic diseases that can cause dependency, and depressive symptoms.^14^

The high rate of depressive symptoms in institutionalized elderly is a major cause for concern.^15^^,^ 16 A recent review of the literature found improvements in sleep quality of older adults achieved by treating depressive symptoms.^10^

The high prevalence of depressive symptoms and poor sleep quality are a concern given the negative impact on the health and well-being of institutionalized elderly. Therefore, the objective of the present study was to compare the sleep profile and depressive symptoms of elderly nursing home residents.

## METHODS

### Participants

A quantitative descriptive study was conducted of elderly residents from two different nursing homes: 4 facilities in the eastern part and 2 in the southern region of São Paulo city. Inclusion criteria were: have preserved cognition and no serious language or comprehension limitations preventing normal application of the data collection instruments. To this end, residents’ medical records containing a full description of the new resident’s state of health were analyzed. An interview was conducted with the manager responsible for maintaining the medical records who was aware of the evolution of cognitive decline since the resident’s admission.

The convenience sample was selected from lists provided by managers of each nursing home in which the elderly met the inclusion criteria, yielding an initial group of 38 participants. Of this group, 5 were subsequently excluded for cognitive impairment, 1 due to death, 1 for having left the institution and 2 for taking part in the pilot stage. This gave a final study sample of 29 elderly.

All ethical norms governing research involving humans were observed. The study was approved by the management of the nursing homes and approved by the Ethics Committee for Research in Humans of the Escola de Artes, Ciências e Humanidades of the Universidade de São Paulo (CAAE no. 38855714.9.0000.5390). Participation was on a voluntary basis and all who took part signed the Free and Informed Consent Form.

### Procedures and measures

The study entailed two stages: pilot and data collection. The pilot stage was carried out to identify possible errors in study planning and to reduce bias in the execution of the procedures envisaged, thereby assuring the validity of the method. The second stage involved data collection by conducting individual interviews at the nursing home facilities. The interviews were held in single sessions each lasting around 35 mins conducted between February 2015 and August 2015. The place of interview was the resident’s room, when individual, because there was a desk and two chairs; for residents who did not have individual rooms, the external area of ​​the institution, where there was a lunch table, was used. In general, there were no interruptions or major noise that could affect the attention state of the participant. This same procedure was followed for all institutions.

The sociodemographic data were gathered by a questionnaire devised by the researchers collecting information on gender, age, education, marital status and previous living companion.

Sleep was assessed using a Sleep Diary composed of 7 questions on sleep latency, number of nighttime awakenings, wakeup method used and daytime napping. Chronotype was determined by applying the Morningness-Eveningness Questionnaire comprising 19 questions attributing a value to each answer, with a total score ranging from 16 to 86 points. Individuals scoring >58 points are classified as morning types, <42 as evening types and 42-58 as intermediate/indifferent types.^17^

Depressive symptoms were assessed using the 15-item Geriatric Depression Scale (GDS), scored as the sum of all scores.^18^ In the present study, total score was classified as no depression signs (1-4 points), dysthymia state (5-6 points) and presence of major depression (7-15 points).

### Statistical analysis

The data were keyed into Microsoft Excel and statistical analyses were performed using computer software programs SPSS v.17.0 and Statistics v. 7.0.

Demographic, sleep and depressive symptoms data were expressed as frequency, percentage, mean and standard deviation. Normality tests revealed that chronotype and GDS-15 scores had a normal distribution, whereas the variable for sleep times had a non-normal distribution. Therefore, Student’s *t*-test and the Mann-Whitney U-test were used for comparisons. Data were keyed into the Excel Office 2010 application and subsequently analysed using the statistics software package Statistica v.7.0. The level of significance adopted for the statistical tests was 5%, i.e., p-value <0.05.

## RESULTS

Table 1 shows the sociodemographic characteristics of the 29 elderly nursing home residents. The sample comprised individuals that were predominantly female (72%), aged 80-90 years (48%), widowed (66%), low-educated (0-5 years) (83%) and lived alone prior to institutionalization (52%).

**Table 1:** Sociodemographic characteristics of the elderly nursing home residents (n=29). São Paulo, 2016.

Variables		N	%
Gender	Female	21	72%
	Male	8	28%
Marital status	Widowed	19	66%
	Single	4	14%
	Separated	1	3%
	Not stated	1	3%
	Divorced	1	3%
	Married	3	10%
Age	75-80	3	10%
	80-90	11	48%
	90-99	10	42%
Education	0-5	24	83%
	5-10	2	7%
	10-15	1	3%
	15-20	1	3%
	Not stated	2	7%
Previous living companion	Son/daughter	3	10%
	Someone else	5	17%
	Spouse only	4	14%
	Alone	15	52%
	Not stated	2	7%

With regard to the sleep diary, entries showed that women slept and awoke later than the men. Regarding chronotype, mean score for the women was 68.2 (±3.1) points, classifying this group as evening type. Man had a mean score of 57.6 (±6.0) points, indicating a classification of intermediate/indifferent type.

Most of the elderly exhibited symptoms of major depression (48%; n=14), followed by dysthymia state (28%; n=8) and no signs of depression (24%; n=7). Notably, compared to the men, the women had more depressive symptoms in both dysthymia and major depression categories.

**Figure 1:**
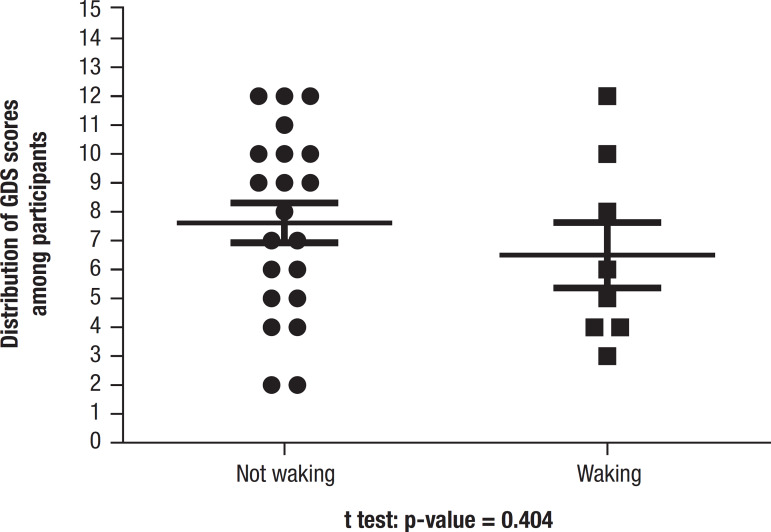
Distribution of GDS scores among participants in different groups (waking or not waking at night).

Comparison of GDS score for nighttime awakening or not is depicted in Figure 1.The GDS scores of individuals who awoke at night or not were similar, with no significant difference between the two groups.

**Figure 2:**
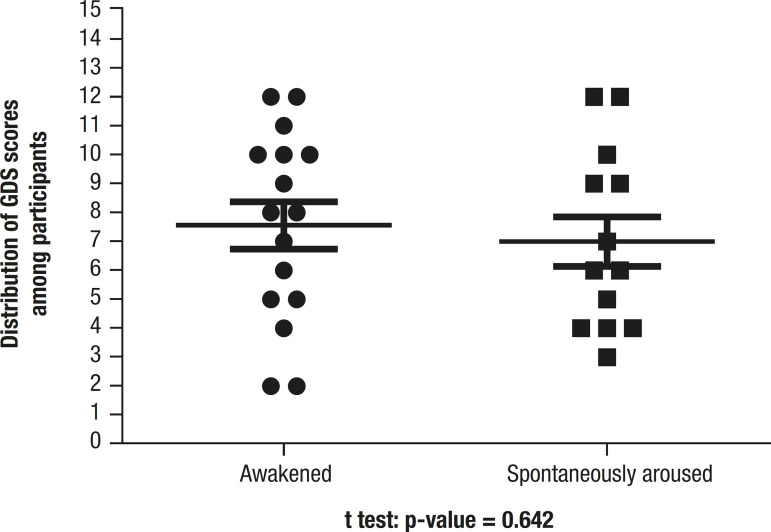
GDS score among participants who were awakened or spontaneously aroused.

Comparison of mean GDS scores for participants who were awoken by disruption or awoke spontaneously is depicted in Figure 2. Again, scores proved similar between the two groups.

**Figure 3:**
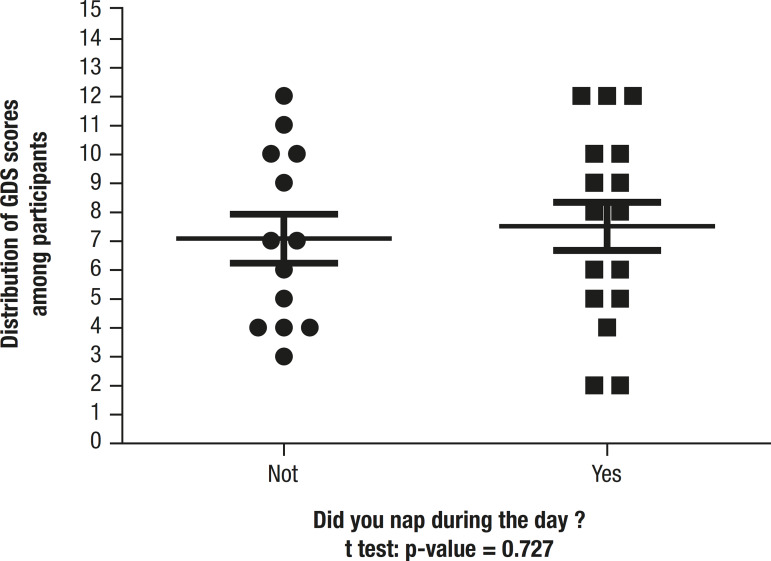
GDS score among participants from different groups.

Comparison of GDS scores for participants who took daytime naps or not is depicted in Figure 3. Results showed no statistically difference between the groups.

**Figure 4:**
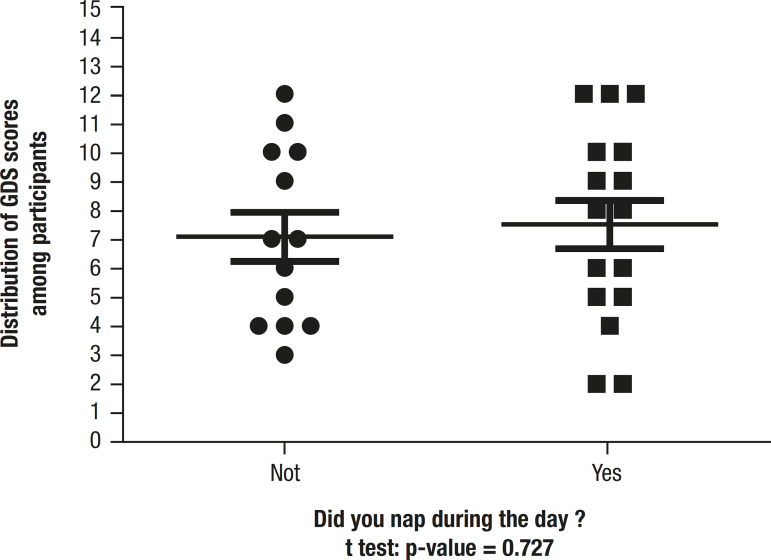
GDS score among participants from different groups according to chronotype test score.

GDS scores for the different groups on the chronotype test are given in Figure 4. Although not reaching statistical significance, the elderly classified as intermediate chronotype had lower scores on the depressive symptoms scale.

## DISCUSSION

The sociodemographic characteristics of the elderly nursing home residents assessed proved similar to those reported in the literature, with participants predominantly female, widowed, low-educated and aged >80 years.^3^^,^ 19 ^,^ 20 The predominance of women can be explained by the process of feminization of old age, attributed to greater self-care throughout life and also lower exposure to external risks compared with men.^21^ Women are often widowed earlier and experience economic hardship, which may predispose them to institutionalization.^22^

The predominantly low educational level reflects the scenario common to developing countries such as Brazil, particularly difficulties accessing school in the past, when education was not considered a priority.^23^ The literature shows variation in the age of institutionalized elderly, but the large number of residents that are widowed or without a companion lends credence to the notion that a weak social and/or family support network is a determinant of institutionalization.^24^

With regard to sleep pattern in the present study, men slept and awoke earlier than women, although this difference was not significant. Regarding chronotype, the women were classified as evening type and men as intermediate/indifferent types.

A number of aging-related changes can influence sleep, such as increased latency, reduced efficacy, greater disruptions, early awakening, shortening deep sleep stages and circadian rhythm sleep-wake disorders.^25^ Elderly nursing home residents tend to exhibit changes in nighttime sleep and circadian rhythm sleep-wake cycle, associated with the aging process, at a higher rate or severity compared to community-dwelling elderly^6^^,^ 8 Sleep disturbances are also more common and severe in nursing home residents than in community-dwelling elderly.^13^^,^ 26

Spending long days in an unstimulating environment without time-reference information or night/day contrasts can lead to irregular sleep cycle patterns and decline in sleep quality, and may also exacerbate existing disturbances. Nursing homes typically have these characteristics, with monotonous routines and low stimulus throughout the day, while nights are disturbed by lights due to the presence of staff in the dormitories and sounds of different events.^6^

In the present study, most elderly had symptoms of major depression. These findings corroborate data in the literature, showing a high prevalence of depressive symptoms among elderly in the institutional setting.^12^^,^ 13 ^,^ 16 It is noteworthy that abandonment is one of the leading causes of depressive symptoms among institutionalized elderly.^13^ A study analyzing the association between depressive symptoms and abandonment in 21 institutionalized elderly found that 80% of the residents had depressive symptoms, of whom 17% were women and 83% men.^27^

Comparisons of GDS score and different sleep measures revealed no statistically significant differences, although elderly classified as intermediate chronotype had lower scores on the depressive symptoms scale. Depressed mood, presence of depressive symptoms and low sense of life satisfaction are associated with poor sleep quality. Therefore, interventions targeting these symptoms in institutional elderly are recommended to promote improvements in sleep and quality of life.^12^

These findings show that male nursing home residents slept and awoke earlier than women. Regarding chronotype, the women were classified as evening types and men as intermediate/indifferent types. Also, the majority of institutionalized elderly exhibited symptoms of major depression.

Study limitations included the cross-sectional design, which precluded the determination of time precedence of the factors studied and drawing of conclusions regarding cause-effect over time. In addition, by decision of the institution owner, we had to use local manager’s knowledge in relation to residents. He indicated which elderly would be considered candidates to participate in the study, with the result that 5 were not eligible for inclusion in the study population. In the beginning of the study, we had total access to the venue and rooms, laundry, kitchen, etc. However, the manager assisting was subsequently dismissed. Thereafter, access became more restricted, limiting the analysis. These results highlight the need for further investigations in this area to inform adequate care planning and help implement interventions in nursing homes aimed at reducing health issues and promoting well-being and quality of life.
